# A High-Throughput Method to Examine Protein-Nucleotide Interactions Identifies Targets of the Bacterial Transcriptional Regulatory Protein Fur

**DOI:** 10.1371/journal.pone.0096832

**Published:** 2014-05-08

**Authors:** Chunxiao Yu, Carlos A. Lopez, Han Hu, Yu Xia, David S. Freedman, Alexander P. Reddington, George G. Daaboul, M. Selim Ünlü, Caroline Attardo Genco

**Affiliations:** 1 Department of Medicine, Section of Infectious Diseases, Boston University School of Medicine, Boston University, Boston, Massachusetts, United States of America; 2 Department of Electrical and Computer Engineering, Boston University, Boston, Massachusetts, United States of America; 3 Department of Microbiology, Boston University School of Medicine, Boston University, Boston, Massachusetts, United States of America; 4 Department of Biomedical Engineering, Boston University, Boston, Massachusetts, United States of America; 5 Bioinformatics Graduate Program, Boston University, Boston, Massachusetts, United States of America; 6 Physics Department, Boston University, Boston, Massachusetts, United States of America; Niels Bohr Institute, Denmark

## Abstract

The Ferric uptake regulatory protein (Fur) is a transcriptional regulatory protein that functions to control gene transcription in response to iron in a number of pathogenic bacteria. In this study, we applied a label-free, quantitative and high-throughput analysis method, Interferometric Reflectance Imaging Sensor (IRIS), to rapidly characterize Fur-DNA interactions *in vitro* with predicted Fur binding sequences in the genome of *Neisseria gonorrhoeae*, the causative agent of the sexually transmitted disease gonorrhea. IRIS can easily be applied to examine multiple protein-protein, protein-nucleotide and nucleotide-nucleotide complexes simultaneously and demonstrated here that seventy percent of the predicted Fur boxes in promoter regions of iron-induced genes bound to Fur *in vitro* with a range of affinities as observed using this microarray screening technology. Combining binding data with mRNA expression levels in a gonococcal *fur* mutant strain allowed us to identify five new gonococcal genes under Fur-mediated direct regulation.

## Introduction

The Interferometric Reflectance Imaging Sensor (IRIS) is a photometric biosensing technology, which is designed on the principles of interferometry and has been applied as a microarray-based screening technique for studying the interactions of biomacromolecules [1], [2], [3], [4]. IRIS can be broadly applied to characterize many types of biomolecular interactions, such as DNA-DNA hybridization, protein-protein binding, and protein-DNA interaction in a quantitative format [1], [2], [3], [4]. Specifically, this technique has previously demonstrated utility for probing transcription factor interactions with arrayed oligonucleotides [5]. Bacterial transcription factors control tight regulation of gene expression in response to host specific environmental niches in a number of human pathogenic bacteria. One such pathogen is *Neisseria gonorrhoeae*, which is the causative agent of the sexually transmitted disease gonorrhea, one of the most common infectious diseases worldwide.

Gonococcal colonization of mucosal surfaces requires tight regulation of gene expression in response to host specific environmental niches including iron-limited host environments. This adaptation is mainly achieved by the up-regulation of iron-acquisition components, which are repressed by a ubiquitous bacterial regulatory protein, the Ferric Uptake Regulator protein (Fur) [6], [7], [8]. Fur is a conserved 15–17 kDa protein comprised of an amino-terminal DNA binding domain and a carboxyl-terminal dimerization domain and is present in a number of diverse bacterial pathogens [9], [10], [11], [12]. In the presence of iron (II) or other divalent cations [9], [10], [11], [13], Fur forms dimers and binds to dsDNA in a sequence specific manner to manipulate transcription initiation [9], [10], [11], [13], [14]. In its most basic form, the iron-bound Fur binds to the -10 and −35 motifs in the promoter region of a gene to prevent binding of RNA polymerase and leads to inhibition of transcription initiation [14]. For example, Fur represses transcription without an iron cofactor, a process termed *apo*-Fur regulation that has been mainly demonstrated in *Helicobacter pylori* [15], [16]. In its *apo*-form Fur has also been reported to function as an activator, auto-regulating the *fur* gene in *Vibrio vulnificus* [17]. The iron-bound Fur dimer has also been reported to function as a transcriptional activator via direct binding to defined promoter regions [8], [18], [19], [20], [21], [22], [23], [24]. Moreover, Fur influences secondary regulatory components, such as small RNAs and histone-like nucleotide binding protein (H-NS), to regulate a subset of genes indirectly [25], [26], [27], [28], [29], [30], [31], [32], [33].

The Fur regulon of *N. gonorrhoeae* has not been well defined due to the lack of a *fur* mutant strain and an efficient and rapid method to characterize the interactions between the gonococcal Fur protein and predicted Fur binding sequences. To define the gonococcal Fur regulon more efficiently, we developed a microarray-based, label-free quantitative method utilizing IRIS to screen sequence-specific interactions of Fur to dsDNA *in vitro* [5], [34]. Combining this approach with bioinformatical analysis of the gonococcal genome and transcriptional analysis of *N. gonorrhoeae* wild type, *fur* mutant and *fur* complemented strains, we identified new Fur regulated genes. We also describe potential additional functional roles for Fur mediated regulation in *N. gonorrhoeae*.

## Materials and Methods

### Prediction of Fur boxes

The sequences upstream (−400 to +50) of ATG, for each open reading frame (ORF) in the *N. gonorrhoeae* FA1090 genome (Genebank No. AE004969.1) were retrieved using RSAT (http://rsat.ulb.ac.be/rsat/) [35] to represent the putative promoter regions of each gene. Seven experimentally determined Fur boxes in the two pathogenic Neisseria species, *N. meningitidis* and *N. gonorrhoeae*, were used as the patterns for Fur box predictions (**Table S1**). Matched sequences in the putative promoter regions were identified using fuzznuc module from EMBOSS by setting the mismatch rate as 0.4 [36]. Each Fur box in **Table S1** provided a list of matched sequences in the entire genome. Subsequently, overlapping sequences across the seven lists were kept as the final output of predicted Fur boxes.

### Interferometric Reflectance Imaging Sensor (IRIS), substituting EMSA

IRIS functions on the principles of interferometry [1], [2], [3], [4], [37]. Briefly, the optical path length of surface-reflected light is affected by changes in accumulated biomass on that surface. Differences in the optical path lengths (OPD) between the top layer and buried silica surface can be measured very precisely, which allows optical height information to be converted to accumulated mass (density) on the surface. With this technique, biomass accumulation can easily be quantified on a surface with a sensitivity of ∼10 pg/mm^2^ for a large field-of-view (FOV) to study multiple binding interactions simultaneously.

### Microarray preparation

Amino-labeled forward DNA strands (50 µM) (Table S2) were mixed with excess complementary strands to create dsDNA through standard hybridization methods: mixed DNA solutions were heated to 80°C for 10 min and then allowed to cool to room temperature in a slow, controlled manner (<1°C/min). IRIS sensor chips were functionalized with a copolymeric surface chemistry to immobilize the amino-functionalized DNA strands in high density [38], [39] as previously demonstrated. The dsDNA solutions were spotted onto the pre-functionalized sensor chips using a non-contact piezo-driven spotter, the sciFLEXARRAYER S3 (Scienion, Inc, Berlin, Germany). After a 12 hr immobilization period, spotted chips were washed with Saline Sodium Citrate (**SSC**) buffer in decreasing concentrations (2x, 0.2x, and 0.1x) to produce a microarray of different covalently-attached dsDNA probes. IRIS images were taken for each chip to determine the initial dsDNA densities corresponding to each spot in the microarray. The chips were then incubated with 0, 200, 400, and 800 nM of purified gonococcal Fur protein [8], [40], [41], [42] in 2 mL of binding buffer (20 mM Tris-Cl pH 7.9, 5 mM MgCl_2_, 40 mM KCl, 0.125 mM MnCl_2_, 2 mM DTT, 10% Glycerol, 0.19 µg/µL poly dIdC), respectively at RT for 2 hr. Subsequently, chips were washed three times (3 min for each wash) using 10 mL binding buffer followed by a 5 sec rinse in 0.1× binding buffer, and images were taken of the post-incubation array.

### Binding interaction analysis

Increases in mass density due to Fur binding were determined by subtracting initial mass densities, determined for each spot from the pre-incubation images, from the final mass density measurements made after incubation [6] (**Figure S1**). On a spot by spot basis, the number of immobilized dsDNA molecules was determined from knowledge of the measured OPD, a known IRIS conversion factor for OPD (nm) to mass surface density (ng/mm^2^) for dsDNA, and the molecular weight of each dsDNA oligomer. For the post-incubation measurements, an increase in the measured OPD was attributed solely to Fur binding and this difference was converted to the number of bound Fur dimers using similar information as described above for calculating the number of immobilized dsDNA probes. In this way, the number of bound Fur dimers could be calculated for each spot within the microarray to effectively determine the number of protein dimers present per dsDNA molecule that was immobilized. For these measurements, between 6 and 10 spots were used to derive statistics on mass density calculations among the same condition.

### Purification of gonococcal Fur

Purification of gonococcal Fur protein was performed as described previously [8]. Briefly, the ORF of the *fur gene* was PCR amplified from genomic DNA of *N. gonorrhoeae* F62 and cloned into a PET15b vector (Novagen, San Diego, CA) by the restriction sites *NdeI* and *BamHI*. The 6xHis-tagged Fur protein was over-expressed in a *E. coli fur* mutant strain, HBMV119 [43] with 0.1 mM IPTG overnight at RT and purified using Ni-charged resin according to the manufacture's protocol (Novagen) [44], [45]. Purified Fur protein was dialyzed against buffer [50 mM Tris-Cl, 500 mM NaCl, 100 µM MnCl_2_, 10% glycerol, pH 7.9]. The 6xHis tag was cleaved using a biotinylated thrombin (Novagen) and removed by flowing through another Ni-charged resin [40], [46], [47].

### Electrophoretic mobility shift assay (EMSA)

The probes used in EMSAs had the same sequences as those probes used in IRIS (**Table S2**). The dsDNA probes were obtained by mixing with its complementary strand at equal concentrations (5 µM), which were subsequently labeled with [γ-^32^P]-ATP using T4 DNA kinase (Applied Biosystems, Ambion, Carlsbad, CA). The radio-labeled probes were purified using a G25 Sephadex QuickSpin column (GE healthcare, Pittsburgh, PA). EMSA was performed as described previously [8], [41], [42]. Labeled dsDNA Probes (12.5 nM) were incubated with purified gonococcal Fur in binding buffer (20 mM Tris-Cl pH 7.9, 5 mM MgCl_2_, 40 mM KCl, 0.125 mM MnCl_2_, 2 mM DTT, 10% Glycerol, 0.19 µg/µL poly dIdC, 3.125 µg/µL BSA) at RT for 30 min. For cold competition assays, unlabeled competitor DNA, as indicated (50 to 1000 fold), was added to the reaction. Each reaction mixture was electrophoresed on a native 6% polyacrylamide gel (acrylamide/bisacrylamide ratio, 37.5∶1 [wt/wt]) (Bio-Rad, Hercules, CA), dried at 80°C on a filter paper for 1 hr and detected by autoradiography. The integrated density of bound and unbound DNA bands was quantified using ImageJ software and the percentage of bound DNA corresponding to a Fur concentration was calculated accordingly in each lane. The binding affinity (KD, equilibrium dissociation constant) of Fur to each DNA probe was calculated using GraphPad Prism Software by plotting the percentage of bound DNA (Y axis) with the Fur concentration (X axis) and using the equation Y =  (Bmax • X)/(KD + X). In this equation, Bmax is the total number of receptors expressed in the same units as the *Y* values.

### RNA purification and quantitative RT-PCR

*N. gonorrhoeae* F62 wild type, *fur* mutant and *fur* complemented strains [48] were plated on GCB agar plates (Remel, Thermo Scientific) and grown overnight at 37°C with 5% CO_2_ and used to inoculate CDM medium [49] containing 0.042% Na_2_CO_3_ with shaking for 2 hr. The cultures were diluted into fresh CDM medium to an OD_600_ = 0.1 and incubated for an additional 2 hr. Subsequently, 100 µM ferric nitrate or 150 µM desferal (deferoxamine mesylate) was added to create iron-replete (+Fe) and iron-deplete (-Fe) conditions, respectively. Bacterial pellets were collected 1 hr after the addition of iron or desferal. RNA was purified using an RNeasy kit (Qiagen, Hilden, Germany) and treated with DNase I. Quantitative RT-PCR was carried out using a One-Step QuantiTect SYBR green RT-PCR kit (QIAGEN) on an ABI Prism 7700 sequence detection system (Applied Biosystems, Foster City, CA) and primers are listed in **Table S4**. A total of 12.5 ng RNA was used in each reaction. The relative mRNA levels of each gene were evaluated using the comparative cycle threshold (ΔΔ*C_T_*) method (Applied Biosystems, *User Bulletin No*. *2: Relative Quantification of Gene Expression*, 1997). The relative expression level of each gene was normalized to the endogenous 16S rRNA gene and represented as the ratio to the expression level of the WT+Fe sample. The experiments were performed four times and results are presented as the mean ± the standard deviation. Statistics was performed using the Student's t test and a *P*<0.05 threshold was considered as significant.

## Results

### Prediction of Fur boxes in the genome of *N. gonorrhoeae* FA1090

Construction of the IRIS chips required a set of double strand DNA probes containing target sequences. To design the dsDNA probes, we first predicted Fur binding sequences (Fur boxes) throughout the genome using a bioinformatics approach. Neisseria Fur boxes previously defined by DNase I foot printing studies were utilized to avoid the mismatches caused by the species-specific nucleotides in Fur boxes of different bacteria (**Table S1**). A total of 198 Fur boxes in the intergenic regions throughout the gonococcal genome were predicted in this study (**Table S5**). Due to the fact that transcription of Fur regulated genes responds to iron availability, we focused on the iron-regulated genes identified via microarray analyses [50], [51] of *N. gonorrhoeae* cultured under iron-replete and iron-deplete conditions. Forty-five iron-repressed genes with predicted Fur boxes in their promoter regions (**Table S3**) were identified, which included 18 out of 21 of the previously identified Fur-repressed genes/operons in *N. gonorrhoeae* [52]. This consistency validated our prediction of Fur binding sequences in the gonococcal genome. Twenty-two Fur binding sequences were predicted in the promoter regions of 21 iron-induced genes in this study (**Table 1**) including 2 previously identified Fur-activated genes, NGO0711 and NGO1751 [8].

**Table 1:** Predicted Fur binding sequences within the promoter regions of *N. gonorrhoeae* iron-induced genes.^1^

Predicted Fur box	Gene	Function	Fur binding	Fur-regulation^2^
TTAAAATATGAATTTAATC	NGO0037	Fe-S oxidoreductases family 1	−4	NE
TATAATCCGCACCGATTTT	NGO0037	Fe-S oxidoreductases family 1	−4	NE
TTAAAATAGAACCATTATC	NGO0073	phosphoglycolate phosphatase	+	NR
TATAAAAAAGAGCATTGTT	NGO0101	cytochrome c4	+	NR
TATTATATAAATTTTTAGC	NGO0155	hypothetical DNA binding protein	+	Repressed
GAAAACAACGATACTTTTC	NGO0302	hypothetical protein	−	NE
TTAATTAACTTTTGTTTTA	NGO0304	phenylalanyl-tRNA synthetase beta subunit	+	NR
CAAAAGAAAAACCGATTTT	NGO0377	NadC family protein, transporter	−	NE
TACACTAAGTATCTTATTT	NGO0436	putative 3-methyl-2-oxobutanoate hydroxymethyltransferase	+	Activated
TATAATAAAATCAATTCTT	NGO0641	type III restriction/modification system modification methylase	+	Repressed
ACAATAAAGTTTCTTTATA	NGO0711^3^	alcohol dehydrogenase	+^4^	NE
TTTAAAAAAAATCAATTTT	NGO0899	transcription elongation factor, GreA	+	NR
TGAAAAAAGAATCCATATC	NGO1189	chaperonin, HslO	+	NR
TAATATCAATATATTGATT	NGO1284	hypothetical protein	+	NR
ACAAAGAAGTATACTTCTT	NGO1419	hypothetical protein	+	NR
TAATATAAGCGGCGGTATT	NGO1684	hypothetical protein	−	NE
CCATACAACTATATTTTTT	NGO1738	NADH dehydrogenase I subunit M	+	Activated
TAACAGCACGCTCATTGTC	NGO1745	NADH dehydrogenase I subunit G	−	NE
TCAAATAAGAATCGTTATC	NGO1751^3^	NADH dehydrogenase I subunit A	+^4^	NE
AAGAAAAAAGATGATTTTC	NGO1845	30S ribosomal protein S12	+	NR
TATAAAAAGAAAGTATTTT	NGO1948	hypothetical protein	+	NR
TAAAAGAAAACTCATTCTC	NGO1957	putative export protein	+	Repressed

1 Iron regulation as previously determined by microarray analyses of *N. gonorrhoeae* grown in defined medium CDM (-Fe) or CDM with 10 µM ferric nitrate (+Fe) [50], [51].

2 Fur regulation was determined by comparing mRNA levels in *N. gonorrhoeae* WT, *fur* mutant and *fur* complemented strains grown under iron-replete and iron-deplete conditions at 1h after addition of iron and desferal using qRT-PCR. NR; not regulated by Fur. Activated; mRNA level of the gene was decreased in the *fur* mutant strain under either iron-replete or iron-deplete conditions compared to that in the wild type strain. Repressed; mRNA level of the gene was increased in the *fur* mutant strain under either iron-replete or iron-deplete conditions compared to that in the wild type strain. NE, not examined; transcriptional regulation was not tested in this study.

3 Fur regulation of the genes as previously determined [8].

4 *In vitro* Fur binding to the 500 bp upstream sequence of ATG of these genes was determined using EMSA and foot printing in previous studies [8].

### Interactions of Fur with predicted consensus sequences in *N. gonorrhoeae*

**Figure 1:**
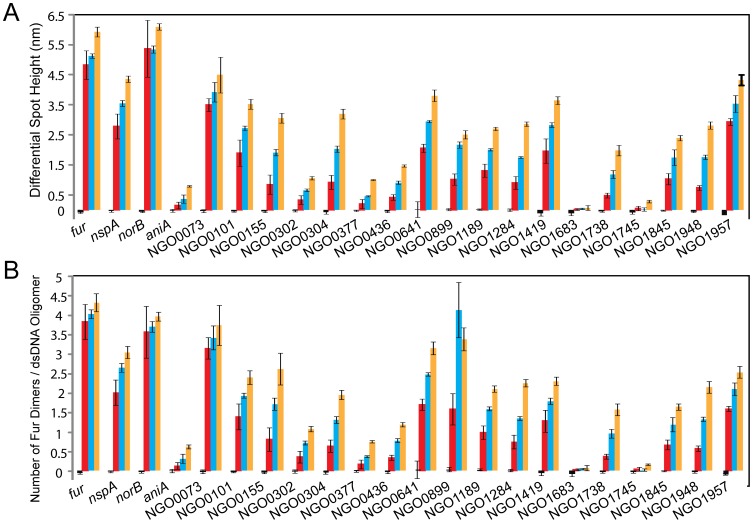
Identification of Fur binding to predicted Fur boxes by label-free IRIS screening. A set of ∼50 bp dsDNA probes containing the predicted Fur box in the middle of the probes were designed for IRIS screening (**Table S4**). Each dsDNA probe was immobilized on a chip to produce a spot with a diameter of approximately 100 µm. Three concentrations of Fur protein, 200 nM (red bars), 400 nM (blue bars) and 800 nM (orange bars), were incubated with the individually prepared arrays (in addition to a 0 nM control incubation (black bars) and the binding of Fur protein to dsDNA spots was measured. A mass increase for each spot was represented as a differential spot height (DSH). The known Fur boxes in *fur*, *norB* and *nspA* promoter regions were used as positive controls, and the *aniA* promoter region was used as negative control in this IRIS assay. (**A**) Differential spot height for each dsDNA probe after incubation with 0 nM, 200 nM, 400 nM and 800 nM Fur protein, respectively. (**B**) The number of Fur dimers bound per dsDNA molecule of each probe as calculated from initial and post-incubation mass density measurements. The gene designations of *N. gonorrhoeae* F62 were assigned according to their homologues in *N. gonorrhoeae* FA1090.

We next characterized *in vitro* binding of the Fur protein to predicted Fur boxes in the promoter regions of iron-induced genes using our high-throughput, label-free technology-IRIS. As shown in **Figure 1A**, Fur binding to the probes containing a predicted Fur box demonstrated a concentration dependent increase in differential spot height (DSH). The Fur boxes in the promoter regions of *fur*, *norB* and *nspA*, which have different binding affinities to Fur (26.1 nM, 1.2 nM and 724.8 nM, respectively) were utilized as positive controls and the sequences in the *aniA* promoter as a negative control [8]. EMSA previously demonstrated that while the *aniA* gene contains a predicated Fur box, the Fur protein does not bind to this region [8]. Consistently, Fur binding to the known Fur boxes in *fur* and *norB* promoter regions reached 4.8±0.5 and 5.4±0.9 nm increase in differential spot height, respectively, when 200 nM Fur protein was added **(** **Figure 1A**). These mass density increases corresponded to 3.8 and 3.6 Fur dimers binding to one dsDNA molecule, respectively (**Figure 1B**). The differential spot height of the *nspA* Fur box probe continued to increase with Fur protein concentrations from 2.8±0.4 nm, 3.5±0.1 nm to 4.4±0.1 nm (**Figure 1A**). Two Fur dimers, 2.6 dimers and 3.0 dimers/*nspA* Fur box were detected when 200 nM, 400 nM and 800 nM Fur protein were added to the chips, respectively (**Figure 1B**). As the negative control, the *aniA* promoter sequences showed a small mass density increase for the three concentrations of Fur protein (DSH<1 nm) (**Figure 1A**). Therefore, we defined a threshold value for a DSH of ≥1 nm as a cutoff for determining specific Fur binding to dsDNA probes.

According to the above analytical criteria, a large number of tested predicted Fur boxes (14 out of 18) in the promoter regions of iron-induced gonococcal genes displayed specific binding properties to the Fur protein, while 4 predicted Fur boxes failed to bind to Fur. Specifically, NGO1683 and NGO1745 did not show increased DSH for any concentration of Fur protein (**Figure 1A**). The DSHs of predicted Fur boxes in NGO302 and NGO0377 promoter regions were only ∼1 nm (1.0±0.06 nm and 1.0±0.02 nm, respectively) when 800 nM Fur protein was added (**Figure 1A**), suggesting that these two predicted sequences did not bind to Fur according to the defined criteria. In contrast, the other 14 predicted Fur boxes showed variable binding properties to Fur. The predicted Fur boxes in the NGO0436 and NGO1738 promoter region displayed DSH values of 1.4±0.05 nm and 2.0±0.2 nm, respectively when 800 nM Fur was added (**Figure 1A**), corresponding to 0.8 dimer/dsDNA molecule and 1.6 Fur dimers/dsDNA molecule, respectively (**Figure 1B**). When 400 nM Fur was added, the predicted Fur boxes in the promoter regions of NGO1284, NGO1845 and NGO1948 showed DSH values of 1.7±0.03 nm, 1.7±0.3 nm and 1.7±0.07 nm, respectively, which all corresponded to 0.5 Fur dimers/dsDNA molecule (half amount of dsDNA is bound by Fur dimers) (**Figure 1A and B**). The Fur boxes in the promoter regions of NGO0155, NGO0304, NGO0899 and NGO1189 had DSH values of 1.9±0.1 nm, 2.0±0.1 nm, 2.2±0.1 nm and 2.0±0.03 nm, respectively (**Figure 1A**). These DSH values corresponded to approximately one Fur dimer/dsDNA molecule (**Figure 1B**). When 200 nM Fur was added, the predicted Fur boxes in NGO0101, NGO0641 and NGO1419 promoter regions had DSH values of 1.9±0.4 nm, 2.0±0.1 nm and 1.9±0.4 nm, respectively which correlated to one Fur dimer/dsDNA (**Figure 1A and B**). The two highest binding affinities were observed for predicted Fur boxes in NGO0073 and NGO1957 promoter regions. When 200 nM Fur was added, DSH values of these two Fur boxes were 3.5±0.4 nm and 2.9±0.1 nm, respectively, corresponding to 3 Fur dimers and 1.5 Fur dimers/dsDNA molecule, respectively (**Figure 1A and B**).

**Figure 2:**
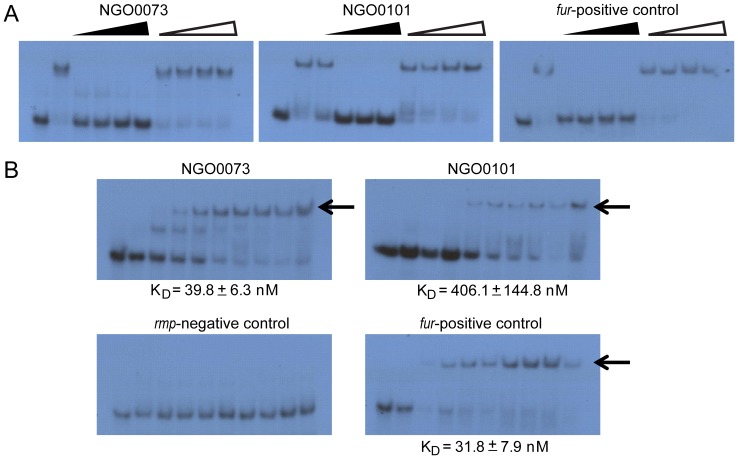
Fur binding to the predicted Fur boxes. (A) Cold competition assay for the specificity of Fur binding to the predicted Fur boxes. ^32^P-labeled ∼50 bp dsDNAs were analyzed after incubation with Fur and unlabeled dsDNA probes (cold probes). Two types of cold competitor probes were used: *fur*, which contains a Fur box, and *rmp*, which does not bind to Fur. When the unlabeled probes *fur* compete out binding of labeled probes, but the unlabeled *rmp* cannot, the binding of Fur to the predicted Fur box was considered specific. Lane 1, free ^32^P-labeled DNA; Lane 2 through Lane 10 contained gonococcal Fur. For the *fur* and NGO0073 Fur box, Fur protein was added at a concentration of 100 nM and for the NGO0101 Fur box, 400 nM of Fur protein was added. The fold excess of the cold probes was increased from Lane 3 to Lane 6 and Lane 7 to Lane 10 ranging from 50 fold, 500 fold to 1000 fold (indicated by the triangles). **(B) Fur binding affinities determined by EMSA.** The ^32^P labeled dsDNA probes were incubated with a gradient of concentrations of purified gonococcal Fur protein (Lane 1 to Lane 10, 0 nM, 5 nM, 25 nM, 50 nM, 100 nM, 200 nM, 400 nM, 600 nM, 800 nM and 1000 nM, respectively). Arrows indicate the shift of Fur-bound probes. The apparent binding affinities (K_D_) were calculated using GraphPad Prism and were represented as mean ± standard error. The gene designations of *N. gonorrhoeae* F62 were assigned according to their homologues in *N. gonorrhoeae* FA1090.

To further validate Fur binding properties of the predicted Fur boxes characterized with IRIS, we performed traditional EMSA on two of the newly identified Fur bound probes, NGO0073 and NGO0101. Fur binding to the predicted Fur boxes in NGO0073 and NGO0101 promoter regions was specific as determined by a cold competition assay (**Figure 2A**). Consistent with the binding affinity trend estimated using IRIS, the apparent K_D_'s of Fur binding to Fur boxes of *fur* gene, NGO0073 and NGO0101 calculated using EMSA results were 31.8±7.9 nM, 39.8±6.3 nM and 406.1±144.8 nM, respectively (**Figure 2B**). The combination of these results supports IRIS as an accurate method to determine the specificity and affinity of Fur binding to an array of immobilized dsDNA sequences.

Of interest among the 18 tested predicted Fur boxes in the promoter regions of iron-induced genes, fourteen were shown to bind to Fur specifically *in vitro* (**Table 1**). No information about Fur binding to these promoter regions was known previously, highlighting the power of IRIS in identifying novel promoters bound by Fur.

### New targets directly regulated by Fur

**Figure 3:**
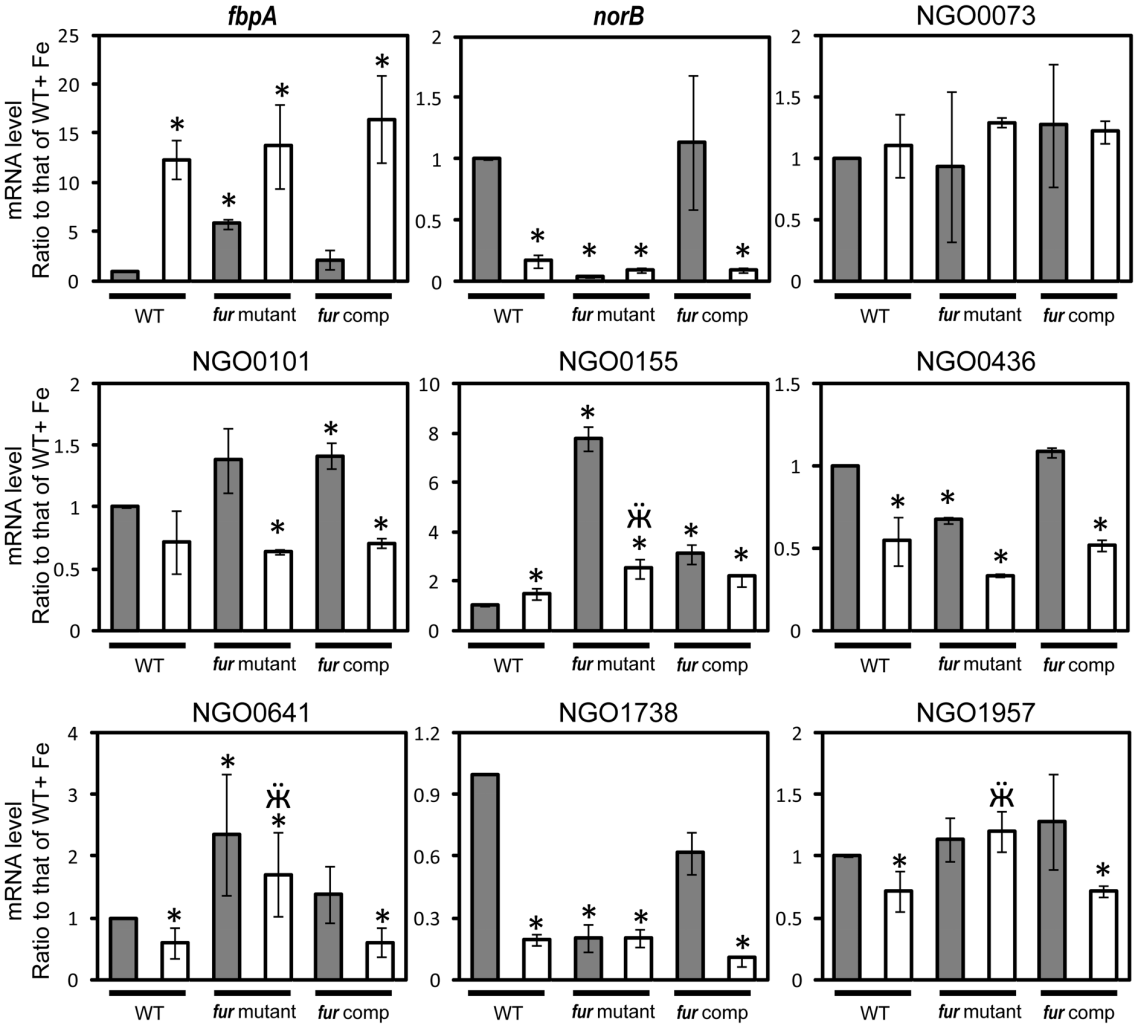
Transcriptional regulation patterns of genes determined by quantitative real-time PCR. The RNA samples were purified from cultures of the wild-type (WT) and *fur* mutant and *fur* complemented strains under iron-replete (+Fe, grey bars) or iron-deplete (-Fe, white bars) conditions 1 h after addition of 100 µM iron or 150 µM desferal. The mRNA levels of *fbpA* and *norB*, genes that were repressed and activated by iron-bound Fur respectively, were used as controls for iron and Fur regulation in *N. gonorrhoeae*. The mRNA levels observed for the five conditions (WT strain under −Fe conditions, *fur* mutant strain under +Fe and −Fe conditions, and *fur* complemented strain under +Fe and −Fe conditions) were compared to the value of WT strain under +Fe conditions. The final results were represented as mean ± standard deviation. A * indicates significantly different compared to the mRNA level of WT+Fe. A ** indicates significantly different compared to the mRNA level of WT-Fe. The gene designations of *N. gonorrhoeae* F62 were assigned according to their homologues in *N. gonorrhoeae* FA1090.

We next examined the regulatory role of Fur in transcription of iron-induced genes containing a functional Fur box in the promoter regions using quantitative RT-PCR of *N. gonorrhoeae* wild type, *fur* mutant, and *fur* complemented strains. This analysis revealed 5 new Fur regulated genes [NGO0155, NGO0436, NGO0641, NGO1738 and NGO1957] (**Figure 3** **,** **Table 1**). An iron bound Fur-repressed gene, *fbpA* [6], [53], was used as a control for Fur and iron regulation. We observed a statistically significant increase (5-fold) in *fbpA* transcript levels in the *fur* mutant strain grown under iron-replete conditions as compared to the wild type strain grown under iron-replete conditions. AS expected we observed restoration of Fur mediated repression in the *fur* complemented strain (**Figure 3**). Transcription of NGO0155 was up-regulated in the *fur* mutant strain compared to the wild type strain under iron-replete (+Fe) conditions, and was partially restored in the *fur* complemented strain (**Figure 3**), suggesting that iron-bound Fur directly represses transcription of NGO0155. Transcriptional levels of NGO0436 and NGO1738 were reduced in the *fur* mutant strain compared to those in the wild type strain and partially restored in the *fur* complemented strain grown under iron-replete conditions (**Figure 3**), suggesting that NGO0436 and NGO1738 are directly activated by iron-bound Fur. Transcription levels of NGO0641 and NGO1957 were up-regulated in the *fur* mutant strain compared to the wild type strain under iron-deplete conditions (-Fe) and were restored in the *fur* complemented strain (**Figure 3**). This transcriptional pattern was similar to that previously observed in *apo*-Fur mediated repression [15], [16].

Transcriptional levels of nine genes, NGO0073, NGO0101 (**Figure 3**), NGO0304, NGO0899, NGO1189, NGO1284, NGO1419, NGO1845 and NGO1948, showed no difference in expression among the three strains (**Table 1** **, Figure S2**). This suggests that these genes are not regulated by Fur under the growth conditions in this study. However, due to the fact that Fur specifically binds to promoter regions of these genes with high affinities, we termed this phenomenon “silent Fur binding” in *N. gonorrhoeae*.

## Discussion

IRIS provides a simple microarray screening technique for fast identification of *in vitro* protein-nucleotide interactions, which substitutes for onerous gel shift assay. This method is able to circumvent complicated and tedious operations such as DNA manipulation that may be hindered due to limited availability of genetic tools for some organisms and can be a complementary method for ChIP-seq [54]. Further application of this microarray screening method includes optimization of biochemical conditions for protein-nucleotide binding, determination of essential nucleotides in the conserved binding sequences, as well as determination of the dissociation constant of a protein-nucleotide interaction through the development of a real-time/dynamic assay.

In this study we used Fur, a global transcriptional protein in *N. gonorrhoeae* to demonstrate the application of IRIS in determining the protein-nucleotide interactions. As a successful application of IRIS in this study, we identified 14 new Fur binding sequences out of 18 tested predicted Fur boxes in the intergenic regions within the gonococcal genome and 5 of these 14 genes were shown to be regulated by Fur under the experimental conditions used (**Figure 3**). NGO0155 encodes a small protein of 4.7 kDa that contains a DNA binding domain. NGO0436 and NGO1738 encode a putative hydroxymethyltransferase and a NADH dehydrogenase I subunit M, respectively, which may be important for the metabolism of *N. gonorrhoeae*. NGO1957 encodes a putative export protein and NGO0641 encodes a DNA methyltransferase (ModA13) of a Type III R-M system. ModA13 methyltransferase recognizes 5′-AGAAA-3′ and methylates the third adenine [55]. Deletion of NGO0641 or phase OFF of NGO0641 in *N. gonorrhoeae* resulted in transcriptional alteration of 17 genes, which suggests that the methyltransferase NGO0641 is involved in epigenetic regulation in *N. gonorrhoeae* [55]. While the relationship between Fur and phase variation needs further investigation, it is plausible that transcriptional repression of NGO0641 by Fur may change the methylation profile of the gonococcal genome and results in epigenetic regulation of the gonococcal transcriptome.

**Figure 4:**
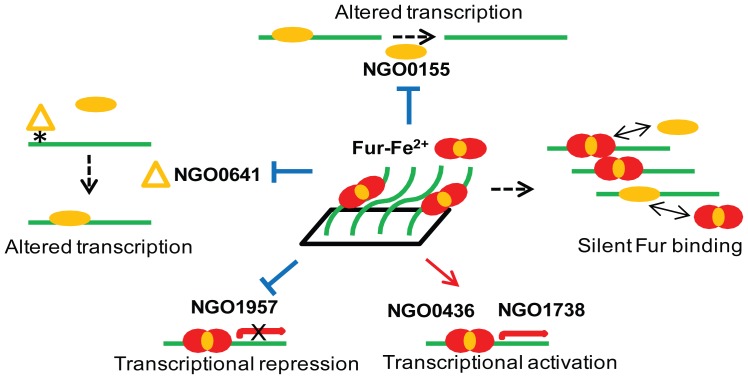
Schematic depicting Fur mediated control mechanisms in *N. gonorrhoeae* as revealed by IRIS. Solid lines with arrowheads indicate direct activation of transcription via Fur binding to promoter regions of gonococcal genes. Solid lines with bars indicate direct repression of transcription via binding of Fur to promoter regions of gonococcal genes. Ellipsoids indicate transcriptional regulatory proteins other than Fur. Transcriptional repression of NGO0641, encoding a methyltransferase (triangle), results in alteration of DNA methylation (*) and subsequently alters transcription of a subset of genes. Transcriptional repression of NGO0155, encoding a putative transcriptional regulator, results in alteration of transcription of NGO0155 target genes. A subset of genes contain a Fur box in their putative promoter regions but were not regulated by Fur under the used growth conditions in this study are termed silent Fur binding.

We also hypothesize a new function for the Fur protein (silent Fur binding), based on the observation that ∼64% of gonococcal genes containing a functional Fur box in promoter regions were not regulated by Fur under the used experimental conditions (**Figure 4**). This phenomenon of silent Fur binding has been recently demonstrated with a novel gonococcal phage repressor protein, Npr [48]. Under *In vivo* conditions, some Fur binding sites may not be accessible due to the binding of other transcriptional regulators and nucleoid-associated proteins including H-NS (histone-like nucleoid structuring protein), IHF (integration host factor), HU (heat unstable protein) and Fis (factor for inversion stimulation) [56]. Thus it is possible that a number of gonococcal genes that contain a Fur box in the promoter regions are not primarily regulated by Fur, but regulated by other transcriptional regulators that may respond to different stimuli. In this scenario, cross-talk of Fur with other regulatory proteins would result in co-regulation of the same targets in response to multiple environmental signals encountered during gonococcal infection. Such condition-specific expression phenomenon was also discovered in the FNR regulon in *E. coli* via a genome-scale ChIP-seq study [54].

Previous studies on Fur regulated genes have been designed to identify Fur regulated genes first in a *fur* mutant strain followed by *in vitro* Fur binding assays to confirm the direct regulatory role of Fur in gene transcription [8], [24], [57], [58], [59]. By design, this type of analysis identifies genes that are either directly or indirectly regulated by Fur. In contrast, IRIS allows for the rapid identification of Fur binding DNA sequences *in vitro* first, followed by subsequent transcriptional analyses. IRIS microarray screening of protein-nucleotide interaction will be a powerful high-throughput tool to complement transcriptome analysis for the identification of regulatory networks of a broad array of bacterial transcriptional proteins.

## Supporting Information

Figure S1 **Mass density image of microarray surface produced using IRIS.** (**Left**) Following incubation with Fur protein (800 nM), an image of the oligonucleotide array is produced quantifying the surface mass density across the entire surface. By comparing the post-incubation densities to those of the pre-incubation image, mass changes attributed to Fur binding can be easily determined on a spot by spot basis. (**Right**) The differential spot heights (DSHs) were determined from the mean of between 500 and 800 total pixels, depending on spot size, used for comparison of the circular spot region (green area) and an outer background annulus region (red region). Pixels in the image which returned large residual error during the data fitting process (ex: salt residue left in the center of each spot – shown here as missing pixels within the green spot region) were eliminated using a threshold value.
(TIF)

Figure S2 **Transcriptional regulation patterns of genes determined by quantitative real-time PCR**. The RNA samples were purified from cultures of the wild-type (WT), *fur* mutant and *fur* complemented strains under iron-replete (+Fe, grey bars) or iron-deplete (-Fe, white bars) conditions1 h after addition of 100 µM iron or 150 µM desferal. The mRNA levels observed for the five conditions (WT strain under −Fe conditions, *fur* mutant strain under +Fe and −Fe conditions, and *fur* complemented strain under +Fe and −Fe conditions) were compared to the value of WT strain under +Fe conditions. The final results were represented as mean ± standard deviation. A * indicates significantly different compared to the mRNA level of WT+Fe. The gene designations of *N. gonorrhoeae* F62 were assigned according to their homologues in *N. gonorrhoeae* FA1090.
(TIF)

Table S1 **Experimentally determined Neisserial Fur boxes used as templates for prediction of Fur boxes in the genome of** ***N. gonorrhoeae*** **.**
(DOCX)

Table S2 **Predicted Fur boxes in the promoter regions of** ***N. gonorrhoeae*** **iron-repressed genes.**
(DOCX)

Table S3 **Double strand DNA probes used in IRIS.**
(DOCX)

Table S4 **Primers used for quantitative RT-PCR.**
(DOCX)

Table S5 **Predicted Fur boxes in the genome of** ***N. gonorrhoeae*** **.**
(DOCX)
