# Supplementary figures and images for: A High-Throughput Method to Examine Protein-Nucleotide Interactions Identifies Targets of the Bacterial Transcriptional Regulatory Protein Fur
Source: PLoS One. 2014 May 8;9(5):e96832. doi: 10.1371/journal.pone.0096832 (PMC4014563; doi:10.1371/journal.pone.0096832)

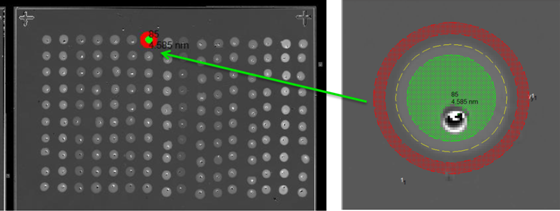

Supplement: Figure S1 — Mass density image of microarray surface produced using IRIS. (Left) Following incubation with Fur protein (800 nM), an image of the oligonucleotide array is produced quantifying the surface mass density across the entire surface. By comparing the post-incubation densities to those of the pre-incubation image, mass changes attributed to Fur binding can be easily determined on a spot by spot basis. (Right) The differential spot heights (DSHs) were determined from the mean of between 500 and 800 total pixels, depending on spot size, used for comparison of the circular spot region (green area) and an outer background annulus region (red region). Pixels in the image which returned large residual error during the data fitting process (ex: salt residue left in the center of each spot – shown here as missing pixels within the green spot region) were eliminated using a threshold value. (TIF) [file pone.0096832.s001.tif]

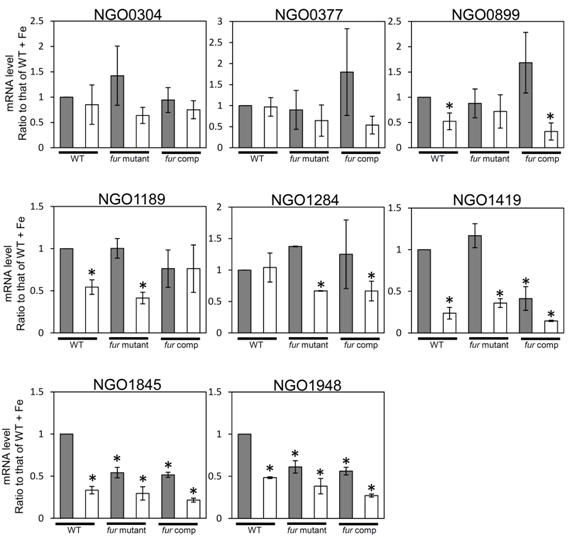

Supplement: Figure S2 — Transcriptional regulation patterns of genes determined by quantitative real-time PCR. The RNA samples were purified from cultures of the wild-type (WT), fur mutant and fur complemented strains under iron-replete (+Fe, grey bars) or iron-deplete (-Fe, white bars) conditions1 h after addition of 100 µM iron or 150 µM desferal. The mRNA levels observed for the five conditions (WT strain under −Fe conditions, fur mutant strain under +Fe and −Fe conditions, and fur complemented strain under +Fe and −Fe conditions) were compared to the value of WT strain under +Fe conditions. The final results were represented as mean ± standard deviation. A * indicates significantly different compared to the mRNA level of WT+Fe. The gene designations of N. gonorrhoeae F62 were assigned according to their homologues in N. gonorrhoeae FA1090. (TIF) [file pone.0096832.s002.tif]
