# Supplementary material for: A High-Throughput Method to Examine Protein-Nucleotide Interactions Identifies Targets of the Bacterial Transcriptional Regulatory Protein Fur
Source: PLoS One. 2014 May 8;9(5):e96832. doi: 10.1371/journal.pone.0096832 (PMC4014563; doi:10.1371/journal.pone.0096832)
Supplement: Table S1 — Experimentally determined Neisserial Fur boxes used as templates for prediction of Fur boxes in the genome of N. gonorrhoeae . (DOCX) [file pone.0096832.s003.docx]

**Table S1. Experimentally determined Neisserial Fur boxes used as templates for prediction of Fur boxes in the genome of *N. gonorrhoeae*.**

| **Gene** | **Fur box** | **References** |
| --- | --- | --- |
| ***N. meningitides*** | | |
| *norB* | GATAATAACTATCATTATT | [[1](#_ENREF_1)] |
| *aniA* (*pan1*) | TATTAGAAGTATCATTTTA | [[1](#_ENREF_1)] |
| *nuoA* | TCAAATAAGAATCGTTATC | [[1](#_ENREF_1)] |
| *nspA* | TATAATACAGCAGGATTCT | [[2](#_ENREF_2)] |
| ***N. gonorrhoeae*** | | |
| *fbpA* | AAATTTAAAAAAATAATTA | [[3](#_ENREF_3)] |
| *fur* | GATAATCATACGCTTAAGC | [[4](#_ENREF_4)] |
| *tonB* | TGCAAATAGGAATTGTTGC | [[4](#_ENREF_4)] |

**References**

1. Delany I, Rappuoli R, Scarlato V (2004) Fur functions as an activator and as a repressor of putative virulence genes in Neisseria meningitidis. Mol Microbiol 52: 1081-1090.

2. Shaik YB, Grogan S, Davey M, Sebastian S, Goswami S, et al. (2007) Expression of the iron-activated nspA and secY genes in Neisseria meningitidis group B by Fur-dependent and -independent mechanisms. J Bacteriol 189: 663-669.

3. Desai PJ, Angerer A, Genco CA (1996) Analysis of Fur binding to operator sequences within the Neisseria gonorrhoeae fbpA promoter. J Bacteriol 178: 5020-5023.

4. Agarwal S, Sebastian S, Szmigielski B, Rice PA, Genco CA (2008) Expression of the gonococcal global regulatory protein Fur and genes encompassing the Fur and iron regulon during in vitro and in vivo infection in women. J Bacteriol 190: 3129-3139.
