# Supplementary material for: A High-Throughput Method to Examine Protein-Nucleotide Interactions Identifies Targets of the Bacterial Transcriptional Regulatory Protein Fur
Source: PLoS One. 2014 May 8;9(5):e96832. doi: 10.1371/journal.pone.0096832 (PMC4014563; doi:10.1371/journal.pone.0096832)
Supplement: Table S2 — Predicted Fur boxes in the promoter regions of N. gonorrhoeae iron-repressed genes. (DOCX) [file pone.0096832.s004.docx]

**Table S2. Predicted Fur boxes in the promoter regions of *N. gonorrhoeae* iron-repressed genes^1^.**

| **Predicted Fur box** | **Gene** | **Function** |
| --- | --- | --- |
| CATATATAATAATCGTTAC | NGO0108^2^ | putative oxidoreductase |
| TTTAAAAAAGAAACTTTGA | NGO0114^2^ | putative glutaredoxin |
| AAATATCAGTATGATTTGC | NGO0142 | putative sugar transporter |
| TAACATAACATTCATTATG | NGO0168 | putative ABC transporter, periplasmic binding protein |
| TATTTTAAAACAAATTATC | NGO0200 | putative phosphoenol pyruvate synthase |
| GCAAATAATTATTTTTTAA | NGO0217^2^ | *fbpC* |
| AAACATAATTACCAGTTTC | NGO0227 | hypothetical protein |
| TATATATACGAATTATATC | NGO0318^2^ | *recN* |
| AAAAAATAGGAACAATTAT | NGO0322^2^ | hypothetical protein |
| TTTTATAACATTCTGTTTT | NGO0328 | hypothetical protein |
| TAATATTCAGATAATTATT | NGO0387 | hypothetical protein |
| TATAAATCGGATTAATGGT | NGO0449^2^ | *sodB* |
| TGCTATAACGGTATTTATA | NGO0461 | hypothetical protein |
| ATTAAATAGCTTCATTATA | NGO0479 | phage-related repressor protein |
| AAAAATAACTATTATTAAT | NGO0553^2^ | *tdfG* |
| TGCAAATAATAATCTAATT | NGO0554^2^ | hypothetical protein |
| TTTTAAAAGCATGGTTATT | NGO0565 | putative pyruvate dehydrogenase E1 component |
| TATAATAAATATCGATAAA | NGO0627 | putative site-specific recombinase |
| TAAAATAAGCAACAATTTT | NGO0629 | DNA gyrase (topoisomerase II) A subunit |
| ATTTAGAACAATCGTCTTT | NGO0633 | putative NifU-like protein |
| GATAATAAAAGTAATTTTC | NGO0652^2^ | Thioredoxin I |
| TATAATAAAACCCCTTATC | NGO0745 | hypothetical protein |
| TGCAAACAATATCATTCTT | NGO0754 | putative molybdopterin-guanine dinucleotide biosynthesis protein |
| TATAATGTCAATATTTTTT | NGO0861 | hypothetical protein |
| AATTATACAAATCATTTTG | NGO0926 | putative peroxiredoxin family protein/glutaredoxin |
| TAACATAACAAACTTTATC | NGO0930 | putative additional 50S ribosomal protein |
| GAATCAAAGATTCCTTATC | NGO0947 | putative dihydrodipicolinate synthase |
| TAAAATAACAAAAATTTAC | NGO0981 | putative peptidyl-prolyl cis-trans isomerase |
| AATAAGTGGATTCATTATA | NGO0988 | putative methylated-DNA-protein-cysteine  methyltransferase |
| TAAAATAATATTCGGTTTT | NGO0994 | lipid modified azurin protein |
| TAAAATAAACAAGCTTTTT | NGO1013 | putative phage repressor |
| TGTGAATAAGAGTGATTCG | NGO1029^2^ | *fumC* |
| GAATATTAAAATCAATTTT | NGO1082 | putative isocitrate dehydrogenase |
| TAAAAGAAAGACTATTCTA | NGO1085 | putative phage associated protein |
| TTTATTATGAATCGTTATT | NGO1158 | hypothetical protein |
| TTTAAATAGAATTTTTATT | NGO1237 | hypothetical protein |
| TATTTTAATTAACGATATT | NGO1291 | putative disulfide bond formation protein |
| GATAATAAATTTCGTTTAT | NGO1318^2^ | *hemO-hemR* |
| TAATATAACATATATTTTT | NGO1442 | putative alcohol dehydrogenase |
| TACAACAAACAACCTTACA | NGO1456 | putative aminopeptidase |
| AATGATAAGGATTATTATT | NGO1496^2^ | *tbpB* |
| TGTTAAAAAAGATTGATGT | NGO1565 | putative nicotinate-nucleotide pyrophosphorylase |
| TAAAATAAGGTAAAGTTTT | NGO2029 | putative ubiquinol--cytochrome c reductase  iron-sulfur subunit |
| AAAAATAATTATTATTATT | NGO2093^2^ | ferric enterobactin receptor |
| CATAATAAGAACCGCTTTT | NGO2109^2^ | *hpuB* |

^1^ Iron regulation as previously determined by microarray analyses of *N. gonorrhoeae* grown in defined medium CDM (-Fe) or CDM with 10 µM ferric nitrate (+Fe) [[1](#_ENREF_1),[2](#_ENREF_2)].

^2^ Fur regulation of the genes as previously determined [[3](#_ENREF_3)].

**References**

1. Ducey TF, Carson MB, Orvis J, Stintzi AP, Dyer DW (2005) Identification of the iron-responsive genes of Neisseria gonorrhoeae by microarray analysis in defined medium. J Bacteriol 187: 4865-4874.

2. Jackson LA, Ducey TF, Day MW, Zaitshik JB, Orvis J, et al. (2010) Transcriptional

and functional analysis of the Neisseria gonorrhoeae Fur regulon. J Bacteriol 192: 77-85.

3. Yu C, Genco CA (2012) Fur-mediated global regulatory circuits in pathogenic

Neisseria species. J Bacteriol 194: 6372-6381.
