# Supplementary material for: A High-Throughput Method to Examine Protein-Nucleotide Interactions Identifies Targets of the Bacterial Transcriptional Regulatory Protein Fur
Source: PLoS One. 2014 May 8;9(5):e96832. doi: 10.1371/journal.pone.0096832 (PMC4014563; doi:10.1371/journal.pone.0096832)
Supplement: Table S3 — Double strand DNA probes used in IRIS. (DOCX) [file pone.0096832.s005.docx]

**Table S3. Double strand DNA probes used in IRIS.**

|  | **Double strand probes used in IRIS*** | **Gene** |
| --- | --- | --- |
| 1F | 5' - 5AmMC6 CTGCTTGAATATTTTATAAAAGCGAACGATAATCATACGCTTAAGCGGT -3' | *fur* |
| 1C | 5' - ACCGCTTAAGCGTATGATTATCGTTCGCTTTTATAAAATATTCAAGCAG -3' |  |
| 2F | 5' - 5AmMC 6TTTCAGTATAATACGGCAGGATTCTTTAACGGATTATTAACAATTTTTCTCCCTA -3' | NGO0233 |
| 2C | 5' - TAGGGAGAAAAATTGTTAATAATCCGTTAAAGAATCCTGCCGTATTATACTGAAA -3' |  |
| 3F | 5' - 5AmMC6 CCGAGTAAAGAATAATGATAGTTATTATCATATATGTATTTGTTCTATA -3' | NGO1275 |
| 3C | 5' - TATAGAACAAATACATATATGATAATAACTATCATTATTCTTTACTCGG -3' |  |
| 4F | 5' - 5AmMC6 AGTTATAAAGTATTAGAAGCGTCATTTTAAGTTCATATTTTATGAATTA -3' | NGO1276 |
| 4C | 5' - TAATTCATAAAATATGAACTTAAAATGACGCTTCTAATACTTTATAACT -3' |  |
| 6F | 5' - 5AmMC6 CTGTATAAATGATAATGGTTCTATTTTAATAAAGCACAACGCGGCTTGT -3' | NGO0073 |
| 6C | 5' - ACAAGCCGCGTTGTGCTTTATTAAAATAGAACCATTATCATTTATACAG -3' |  |
| 7F | 5' - 5AmMC6 CATATTTGAATATAAAAAAGAGCATTGTTGCGTTATCCAATGCTGTAAT -3' | NGO0101 |
| 7C | 5' - ATTACAGCATTGGATAACGCAACAATGCTCTTTTTTATATTCAAATATG -3' |  |
| 8F | 5' - 5AmMC6 GATATTTTGTGCTAAAAATTTATATAATATTTAAATTAATATCAAGTTA -3' | NGO0155 |
| 8C | 5' - TAACTTGATATTAATTTAAATATTATATAAATTTTTAGCACAAAATATC -3' |  |
| 9F | 5' - 5AmMC6 CCGCCTTTTTGAAAACAACGATACTTTTCAGACGGCCTTGAAACCCAAA -3' | NGO0302 |
| 9C | 5' - TTTGGGTTTCAAGGCCGTCTGAAAAGTATCGTTGTTTTCAAAAAGGCGG -3' |  |
| 10F | 5' - 5AmMC6 GAACGAAAATTGATAAAACAAAAGTTAATTAATTGATTGAGAACATAAC -3' | NGO0304 |
| 10C | 5' - GTTATGTTCTCAATCAATTAATTAACTTTTGTTTTATCAATTTTCGTTC -3' |  |
| 11F | 5' - 5AmMC6 TTGCACGATACAAAAGAAAAACCGATTTTGTTGCCTTAAAGGAGCATTC -3' | NGO0377 |
| 11C | 5' - GAATGCTCCTTTAAGGCAACAAAATCGGTTTTTCTTTTGTATCGTGCAA -3' |  |
| 13F | 5' - 5AmMC6 ATTTTGTTTTAAATAAGATACTTAGTGTATTTTTATGAGGGGGTCGAAC -3' | NGO0436 |
| 13C | 5' - GTTCGACCCCCTCATAAAAATACACTAAGTATCTTATTTAAAACAAAAT -3' |  |
| 14F | 5' - 5AmMC6 AAAGACAGCAAAGAATTGATTTTATTATAATTTATTTGTAAAGCCATTT -3' | NGO0641 |
| 14C | 5' - AAATGGCTTTACAAATAAATTATAATAAAATCAATTCTTTGCTGTCTTT -3' |  |
| 17F | 5' - 5AmMC6 TTTTATGCCGAAAATTGATTTTTTTTAAATTTTTTGTTTCTAAAATTTT -3' | NGO0899 |
| 17C | 5' - AAAATTTTAGAAACAAAAAATTTAAAAAAAATCAATTTTCGGCATAAAA -3' |  |
| 18F | 5' - 5AmMC6 ACTGCCGGTTTGAAAAAAGAATCCATATCGGTGTTTCCGCGCAGGAGGC -3' | NGO1189 |
| 18C | 5' - GCCTCCTGCGCGGAAACACCGATATGGATTCTTTTTTCAAACCGGCAGT -3' |  |
| 20F | 5' - 5AmMC6 ACACGGGAAATAATATCAATATATTGATTTACAAACATAAAAATCATGC -3' | NGO1284 |
| 20C | 5' - GCATGATTTTTATGTTTGTAAATCAATATATTGATATTATTTCCCGTGT - 3' |  |
| 21F | 5' - 5AmMC6 TGACGAAAAAACAAAGAAGTATACTTCTTTTTAGTTATGGTTGATTCCA -3' | NGO1419 |
| 21C | 5' - TGGAATCAACCATAACTAAAAAGAAGTATACTTCTTTGTTTTTTCGTCA -3' |  |
| 22F | 5' - 5AmMC6 GCGGCGATTGTAATATAAGCGGCGGTATTTGTGTAGTTTTCTTCAGACG -3' | NGO1683 |
| 22C | 5' - CGTCTGAAGAAAACTACACAAATACCGCCGCTTATATTACAATCGCCGC -3' |  |
| 23F | 5' - 5AmMC6 AAAGACCAACCCATACAACTATATTTTTTATTTTAACCACAGGTTAACC -3' | NGO1738 |
| 23C | 5' - GGTTAACCTGTGGTTAAAATAAAAAATATAGTTGTATGGGTTGGTCTTT -3' |  |
| 24F | 5' - 5AmMC6 TACAGATGAAGACAATGAGCGTGCTGTTAAAGAGTTATTTAAGTCATTT -3' | NGO1745 |
| 24C | 5' - AAATGACTTAAATAACTCTTTAACAGCACGCTCATTGTCTTCATCTGTA -3' |  |
| 25F | 5' - 5AmMC6 CGGCAAACAAGAAAATCATCTTTTTTCTTGTCGTTATGCTTGACTGTCT -3' | NGO1845 |
| 25C | 5' - AGACAGTCAAGCATAACGACAAGAAAAAAGATGATTTTCTTGTTTGCCG -3' |  |
| 26F | 5' - 5AmMC6 TCTTTCCGTCAAAATACTTTCTTTTTATATTCATTAACTTGTTAAATTA -3' | NGO1948 |
| 26C | 5' - TAATTTAACAAGTTAATGAATATAAAAAGAAAGTATTTTGACGGAAAGA -3' |  |
| 29F | 5' - 5AmMC6 AAAAAACACAGAGAATGAGTTTTCTTTTAAAATACAACATTTTTTAACA -3' | NGO1957 |
| 29C | 5' - TGTTAAAAAATGTTGTATTTTAAAAGAAAACTCATTCTCTGTGTTTTTT -3' |  |
| F | TAGTGGAGACTGAAATATCCGATTTGCCGCCATGTTTCTACAGCGGCCTG | *rmp* |
| C | CAGGCCGCTGTAGAAACATGGCGGCAAATCGGATATTTCAGTCTCCACTA |  |

*Underlined sequences are the predicted Fur boxes in this study.
