# Supplementary material for: A High-Throughput Method to Examine Protein-Nucleotide Interactions Identifies Targets of the Bacterial Transcriptional Regulatory Protein Fur
Source: PLoS One. 2014 May 8;9(5):e96832. doi: 10.1371/journal.pone.0096832 (PMC4014563; doi:10.1371/journal.pone.0096832)
Supplement: Table S4 — Primers used for quantitative RT-PCR. (DOCX) [file pone.0096832.s006.docx]

**Table S4. Primers used for quantitative RT-PCR.**

| **Gene** | **Oligonucleotides** | **Length of product** |
| --- | --- | --- |
| NGO0073_RT_F | CCTGTCTGGACAAGCTCAAA | 177 bp |
| NGO0073_RT_R | CGCAGAGTCCGAATACCAT |  |
| NGO0101_RT_F | TCATCAAACTATCGGCATCC | 158 bp |
| NGO0101_RT_R | TTTCCTTAGGATTGGCTTCG |  |
| NGO0155_RT_F | ATCGGATACGGTGAAACGA | 82 bp |
| NGO0155_RT_R | CAAACAATACAGGCTTTCGC |  |
| NGO0302_RT_F | CAGGTACAAGAAATTATTGACTA | 131 bp |
| NGO0302_RT_R | CCCTGAACATATTGCTGTCC |  |
| NGO0304_RT_F | GCCTGATGATGGTGTAGACG | 174 bp |
| NGO0304_RT_R | TGAGTCAACGCAGAAACCTC |  |
| NGO0377_RT_F | CCTACGTCTTCCTCCTGCTC | 160 bp |
| NGO0377_RT_R | GGCAGAATCAACAGCATCAT |  |
| NGO0436_RT_F | CAGAGTAAGGAGCAGGCGTT | 188 bp |
| NGO0436_RT_R | TGAACTTTATATCCGCCGAA |  |
| NGO0641_RT_F | TTTGAAGGTTTATGCGAACG | 188 bp |
| NGO0641_RT_R | CCTGCAAATTGTTCATCGTC |  |
| NGO0899_RT_F | TTTCCGAGTTGGAACACAAA | 193 bp |
| NGO0899_RT_R | ATCGGAGAGCCGACATAGAT |  |
| NGO1189_RT_F | GCGCAAATGTTGGTGAACTA | 152 bp |
| NGO1189_RT_R | AGCGTACTGACGTGTTCCC |  |
| NGO1284_RT_F | TGGTTGAAGACATCGGCTAC | 195 bp |
| NGO1284_RT_R | CGAAGGAAACGGTAACAGTG |  |
| NGO1419_RT_F | CCCGAATACGATGATTGGA | 75 bp |
| NGO1419_RT_R | GCGTTGCCATTTGTTGTAGT |  |
| NGO1683_RT_F | AACGGGCAACTGGATTAAAG | 194 bp |
| NGO1683_RT_R | CTTCCGACCAGTCGGTACTT |  |
| NGO1738_RT_F | ATGCGTTTATTACGCTGCTG | 161 bp |
| NGO1738_RT_R | CAGCATACCCTCGAAGAACA |  |
| NGO1745_RT_F | GAGCCTGAAATCGATACGGT | 165 bp |
| NGO1745_RT_R | ATTGATGAAGCTGCCTGATG |  |
| NGO1845_RT_F | AAACCTAACTCTGCATTGCG | 156 bp |
| NGO1845_RT_R | GTAACGTACACCCGGCAAGT |  |
| NGO1948_RT_F | ATCAGTTTCCATTCCCGTTC | 199 bp |
| NGO1948_RT_R | TAACCGTTGTCGCAGACATT |  |
