# Supplementary material for: A High-Throughput Method to Examine Protein-Nucleotide Interactions Identifies Targets of the Bacterial Transcriptional Regulatory Protein Fur
Source: PLoS One. 2014 May 8;9(5):e96832. doi: 10.1371/journal.pone.0096832 (PMC4014563; doi:10.1371/journal.pone.0096832)
Supplement: Table S5 — Predicted Fur boxes in the genome of N. gonorrhoeae . (DOCX) [file pone.0096832.s007.docx]

**Table S5. Predicted Fur boxes in the genome of *N. gonorrhoeae*.**

| **Fur box** | **Gene** | **Start** | **Ending** | **Strand** | **Mismatch** |
| --- | --- | --- | --- | --- | --- |
| TGAAAATATTATATTTTCT | NGO0001 | 142 | 160 | + | 7 |
| CATATGCAGAATCCTTTTT | NGO0006 | 138 | 156 | + | 9 |
| TAGAAAAATGATTATTGGC | NGO0009 | 13 | 31 | + | 8 |
| AGCAAAAATCAAATGCCGT | NGO0033 | 42 | 60 | - | 8 |
| TTAAAATATGAATTTAATC | NGO0037 | 43 | 61 | - | 8 |
| TGAAAAAAGTATTTATCCG | NGO0037 | 202 | 220 | + | 5 |
| *TATAATCCGCACCGATTTT | NGO0037 | 261 | 279 | + | 9 |
|  | NGO0039 | 88 | 106 | - | 9 |
| TGAAAAAAGTTTTAATCGC | NGO0044 | 118 | 136 | + | 6 |
| TGAAAATAGAGAAATTTAA | NGO0063 | 86 | 104 | - | 7 |
| TATAAATGATAATGGTTCT | NGO0073 | 57 | 75 | + | 8 |
| TTAAAATAGAACCATTATC | NGO0073 | 264 | 282 | - | 9 |
| TATAAAAAAGAGCATTGTT | NGO0100 | 33 | 51 | - | 6 |
|  | NGO0101 | 150 | 168 | + | 6 |
| TGAAAAAGGGAAGTATTGC | NGO0105 | 176 | 194 | - | 7 |
| CATATATAATAATCGTTAC | NGO0107 | 87 | 105 | - | 7 |
|  | NGO0108 | 52 | 70 | + | 7 |
| TAAAAAAAGCAATTATAAG | NGO0107 | 166 | 184 | - | 8 |
| TTTAAAAAAGAAACTTTGA | NGO0113 | 86 | 104 | - | 8 |
|  | NGO0114 | 79 | 97 | + | 8 |
| AAATATCAGTATGATTTGC | NGO0142 | 50 | 68 | + | 8 |
| TGAAAAAAGGCGTACATTC | NGO0155 | 151 | 169 | + | 8 |
| TATTATATAAATTTTTAGC | NGO0155 | 211 | 229 | - | 9 |
| TAACATAACATTCATTATG | NGO0168 | 40 | 58 | - | 9 |
| GGTAATAAATATGGGTTTG | NGO0192 | 67 | 85 | - | 9 |
| TATTTTAAAACAAATTATC | NGO0200 | 52 | 70 | + | 8 |
|  | NGO0201 | 285 | 303 | - | 8 |
| GCAAATAATTATTTTTTAA | NGO0217 | 290 | 308 | - | 8 |
| AAACATAATTACCAGTTTC | NGO0227 | 360 | 378 | - | 9 |
| GAAAACAACGATACTTTTC | NGO0302 | 80 | 98 | + | 9 |
| TTAATTAACTTTTGTTTTA | NGO0304 | 55 | 73 | - | 8 |
| TAAAAATTGGTAATAATCC | NGO0305 | 28 | 46 | + | 8 |
| CGCTAAAAATAATTATTGC | NGO0307 | 42 | 60 | + | 8 |
| TATGATCTGTATCGTATTT | NGO0308 | 153 | 171 | + | 8 |
| TTCAAAAACATATTTTAAC | NGO0313 | 68 | 86 | - | 8 |
| TATATATACGAATTATATC | NGO0318 | 119 | 137 | - | 7 |
| AAAAAATAGGAACAATTAT | NGO0322 | 107 | 125 | + | 7 |
| TTTTATAACATTCTGTTTT | NGO0327 | 117 | 135 | - | 8 |
|  | NGO0328 | 123 | 141 | + | 8 |
| GATAAACAGGCTTTTTTGA | NGO0355 | 213 | 231 | + | 8 |
| AATAATAAAAAATTTTTTA | NGO0367 | 30 | 48 | + | 8 |
| AACACAAAGAATACTTTTT | NGO0371 | 52 | 70 | + | 9 |
|  | NGO0372 | 215 | 233 | - | 9 |
| CAAAAGAAAAACCGATTTT | NGO0377 | 190 | 208 | + | 9 |
| TAATATTCAGATAATTATT | NGO0386 | 15 | 33 | + | 9 |
|  | NGO0387 | 170 | 188 | - | 9 |
| CATAATATGATTCAGTATC | NGO0419 | 87 | 105 | - | 8 |
|  | NGO0420 | 33 | 51 | + | 8 |
| TGAAAATAAAAATAAATCT | NGO0429 | 47 | 65 | - | 8 |
| TTAAACAACTTTATTTTTG | NGO0430 | 159 | 177 | - | 9 |
| TGAAAAAACGCAATAATCC | NGO0433 | 167 | 185 | + | 8 |
| TACACTAAGTATCTTATTT | NGO0435 | 285 | 303 | + | 7 |
|  | NGO0436 | 80 | 98 | - | 7 |
| TGAAAAACGGAAAAAATGG | NGO0448 | 67 | 85 | + | 8 |
| TATAAATCGGATTAATGGT | NGO0449 | 153 | 171 | - | 8 |
| TGCTATAACGGTATTTATA | NGO0461 | 65 | 83 | + | 9 |
| TGAAAATATAACTATTGCC | NGO0475 | 111 | 129 | + | 5 |
| TATAAAAGGCAATAGTTGT | NGO0479 | 178 | 196 | - | 7 |
| TCCAAAAAGCAGTTGATTA | NGO0479 | 101 | 119 | - | 8 |
| ATTAAATAGCTTCATTATA | NGO0479 | 324 | 342 | + | 8 |
|  | NGO0480 | 43 | 61 | - | 8 |
| TGTCCCAATAATCATTAAC | NGO0524 | 49 | 67 | + | 8 |
| AAAAATAACTATTATTAAT | NGO0553 | 90 | 108 | - | 9 |
| CATAATAAGAATTATTTGA | NGO0553 | 144 | 162 | - | 6 |
| TGCAAATAATAATCTAATT | NGO0554 | 83 | 101 | - | 8 |
| ATCGAACACGATTTATTGG | NGO0556 | 38 | 56 | + | 8 |
| TTTTAAAAGCATGGTTATT | NGO0565 | 146 | 164 | + | 7 |
| TTTGATTAGTATGATTTTT | NGO0598 | 2 | 20 | + | 9 |
| TGAAATAAAAGGCATTTTG | NGO0598 | 221 | 239 | + | 7 |
| TGAAAAATGCAATCGTTGT | NGO0614 | 87 | 105 | + | 8 |
| ATCAATACGGATTATTCTA | NGO0627 | 71 | 89 | + | 9 |
|  | NGO0628 | 134 | 152 | - | 9 |
| TATAATAAATATCGATAAA | NGO0627 | 99 | 117 | - | 7 |
|  | NGO0628 | 106 | 124 | + | 7 |
| TAAAATAAGCAACAATTTT | NGO0629 | 56 | 74 | + | 7 |
| ATTTAGAACAATCGTCTTT | NGO0633 | 331 | 349 | + | 9 |
| TATAATAAAATCAATTCTT | NGO0641 | 128 | 146 | - | 8 |
| GATAATAAAAGTAATTTTC | NGO0652 | 125 | 143 | + | 6 |
|  | NGO0653 | 155 | 173 | - | 6 |
| TTCAAAAACGGATTCATCA | NGO0671 | 199 | 217 | - | 8 |
| TTTCAAAAGCATTATTATC | NGO0679 | 187 | 205 | - | 8 |
|  | NGO0680 | 165 | 183 | + | 8 |
| TTAAAAAGTGGATTTATGC | NGO0686 | 187 | 205 | + | 8 |
| TGCAAGAAAGATTTAGTAA | NGO0701 | 38 | 56 | + | 8 |
| AGCAAATATAAAGCATTTG | NGO0703 | 12 | 30 | - | 8 |
| ACAATAAAGTTTCTTTATA | NGO0710 | 40 | 58 | + | 9 |
|  | NGO0711 | 96 | 114 | - | 9 |
| GTCAATAAGAATTATTTTC | NGO0710 | 86 | 104 | + | 8 |
|  | NGO0711 | 50 | 68 | - | 6 |
| GATAAAAATAATGGTTTTT | NGO0736 | 108 | 126 | + | 8 |
|  | NGO0738 | 186 | 204 | - | 8 |
| TATAATAAAACCCCTTATC | NGO0744 | 91 | 109 | - | 9 |
|  | NGO0745 | 35 | 53 | + | 9 |
| CAAATAAACCATCAATTTC | NGO0751 | 294 | 312 | + | 9 |
| GAACAGAAAAATCATTTAC | NGO0751 | 381 | 399 | + | 8 |
| TGCAAACAATATCATTCTT | NGO0753 | 162 | 180 | - | 9 |
|  | NGO0754 | 27 | 45 | + | 9 |
| TCCAAATGATAATCCTTCA | NGO0765 | 145 | 163 | + | 8 |
| TTAAAGTAGTATCTTTCTT | NGO0774 | 120 | 138 | + | 8 |
| ATGAGAAAGATTCATTATA | NGO0794 | 91 | 109 | + | 9 |
| TGAAAAAATCTATTACGGC | NGO0818 | 18 | 36 | + | 8 |
| TTGAAAAGGAAAATGATGG | NGO0847 | 184 | 202 | + | 8 |
| TGAAAACCAGACTGTTTGG | NGO0852 | 174 | 192 | - | 8 |
| TGCAAAAAGATTTTATCCA | NGO0857 | 57 | 75 | - | 8 |
| TATAATGTCAATATTTTTT | NGO0861 | 175 | 193 | - | 7 |
|  | NGO0862 | 48 | 66 | + | 7 |
| TTTAAAAAAAATCAATTTT | NGO0899 | 120 | 138 | - | 7 |
|  | NGO0900 | 253 | 271 | + | 7 |
| GAAATGAAGCATCATAATC | NGO0902 | 41 | 59 | - | 7 |
| AATAAAAACGATACTTTTC | NGO0915 | 210 | 228 | - | 7 |
| AATTATACAAATCATTTTG | NGO0926 | 21 | 39 | + | 9 |
| TAACATAACAAACTTTATC | NGO0929 | 24 | 42 | + | 7 |
|  | NGO0930 | 157 | 175 | - | 7 |
| GAATCAAAGATTCCTTATC | NGO0947 | 79 | 97 | + | 9 |
| TATATTAAAATTCACTTAC | NGO0961 | 215 | 233 | + | 9 |
| TAAAATAACAAAAATTTAC | NGO0981 | 1 | 19 | + | 9 |
|  | NGO0982 | 110 | 128 | - | 9 |
| TACAACGGCAATCCTTTTA | NGO0987 | 64 | 82 | + | 8 |
| TATAATACGCCCCATTCCC | NGO0987 | 176 | 194 | + | 9 |
| AATAAGTGGATTCATTATA | NGO0988 | 190 | 208 | + | 7 |
| TAAAATAATATTCGGTTTT | NGO0994 | 247 | 265 | - | 9 |
| TGCAAAAATAGTCTGTTAA | NGO1000 | 139 | 157 | - | 7 |
| TAAAATAAACAAGCTTTTT | NGO1013 | 38 | 56 | + | 9 |
|  | NGO1014 | 153 | 171 | - | 9 |
| GAAAAGAGAAATATTTTTC | NGO1013 | 178 | 196 | - | 9 |
|  | NGO1014 | 13 | 31 | + | 9 |
| AACAATTAAAACCATTTTT | NGO1024 | 346 | 364 | + | 7 |
| TGTGAATAAGAGTGATTCG | NGO1029 | 107 | 125 | - | 8 |
| TGCCATAAATAATTTTTCA | NGO1032 | 99 | 117 | - | 8 |
| TAGCATAACAAAATTTTTA | NGO1048 | 26 | 44 | - | 9 |
|  | NGO1049 | 148 | 166 | + | 9 |
| CATAAAAAGTATTTTTCTT | NGO1061 | 209 | 227 | + | 6 |
|  | NGO1062 | 174 | 192 | - | 6 |
| TATAAGAAGCAAAGATTGC | NGO1062 | 46 | 64 | + | 8 |
| TTAAAATGGAAATTTGAAC | NGO1067 | 17 | 35 | - | 8 |
| AATAATCATCATCATTCTG | NGO1070 | 369 | 387 | + | 8 |
| TTAAAACAGCCATTGTTTT | NGO1079 | 113 | 131 | - | 7 |
| GAATATTAAAATCAATTTT | NGO1081 | 247 | 265 | - | 8 |
|  | NGO1082 | 108 | 126 | + | 8 |
| TAAAAGAAAGACTATTCTA | NGO1085 | 139 | 157 | + | 9 |
| TGCACAAATTATTTCTTGA | NGO1117 | 89 | 107 | + | 7 |
| TTGATTAAGGATTTTCTGC | NGO1133 | 213 | 231 | + | 8 |
| CAAAATAAAAATCATCTTT | NGO1147 | 101 | 119 | + | 6 |
| TGAAAATCCTCATTTTAGG | NGO1154 | 202 | 220 | + | 8 |
| TTTATTATGAATCGTTATT | NGO1158 | 79 | 97 | - | 7 |
| TTCTATAAGTATGTATAAT | NGO1158 | 217 | 235 | - | 9 |
| AACAATCAATAACATTTTC | NGO1167 | 283 | 301 | - | 7 |
| AATGAAAAGAAGGATTATT | NGO1173 | 24 | 42 | + | 7 |
| TATAAAACGGGTCATTCTA | NGO1178 | 185 | 203 | - | 6 |
| TGAAAAAAGAATCCATATC | NGO1189 | 21 | 39 | + | 9 |
|  | NGO1190 | 242 | 260 | - | 9 |
| TATAATAACAAAATTTTGA | NGO1204 | 2 | 20 | - | 9 |
|  | NGO1205 | 373 | 391 | + | 9 |
| TAAAAGATGAAAGATTTTT | NGO1236 | 179 | 197 | + | 9 |
| TTTAAATAGAATTTTTATT | NGO1237 | 214 | 232 | + | 8 |
| TAATATCAATATATTGATT | NGO1283 | 214 | 232 | - | 8 |
|  | NGO1284 | 117 | 135 | + | 8 |
| TATTTTAATTAACGATATT | NGO1290 | 166 | 184 | + | 7 |
|  | NGO1291 | 143 | 161 | - | 7 |
| GTCAAAAAGCAAGCGTTTT | NGO1293 | 85 | 103 | + | 8 |
| GATTATCAGAAAATTTATT | NGO1317 | 237 | 255 | - | 7 |
| CGCAAATGATATTTTTTCG | NGO1318 | 134 | 152 | + | 8 |
| GATAATAAATTTCGTTTAT | NGO1318 | 240 | 258 | + | 5 |
|  | NGO1319 | 20 | 38 | - | 5 |
| TGCCGAAATATTTTTTTGC | NGO1341 | 133 | 151 | - | 7 |
| TGACAATAATCATATTTCC | NGO1343 | 85 | 103 | - | 8 |
| TGCAAATATTTGTTTTAAC | NGO1360 | 6 | 24 | - | 8 |
|  | NGO1361 | 100 | 118 | + | 8 |
| TATAAAAAAGACTTTTTAT | NGO1366 | 142 | 160 | - | 7 |
| TATAACAATATGATTTATA | NGO1374 | 129 | 147 | + | 9 |
|  | NGO1375 | 167 | 185 | - | 9 |
| TATAAATCATATTGTTATA | NGO1374 | 129 | 147 | - | 9 |
|  | NGO1375 | 167 | 185 | + | 9 |
| TATAATAACGGTCTATATC | NGO1403 | 115 | 133 | + | 7 |
| TAAAATAAATTTATTTTTC | NGO1411 | 68 | 86 | + | 9 |
| ACAAAGAAGTATACTTCTT | NGO1419 | 156 | 174 | + | 8 |
| TAAAAAAACGATATTGATA | NGO1419 | 98 | 116 | - | 8 |
| TTTATATATGAAATGGTTG | NGO1424 | 194 | 212 | + | 8 |
| TTAATATAATATTTTCTGC | NGO1426 | 103 | 121 | - | 8 |
| TAAAACCAATATATTCATA | NGO1429 | 64 | 82 | + | 9 |
| AATAAATAATAATTATTAT | NGO1430 | 169 | 187 | + | 8 |
| TAATATAACATATATTTTT | NGO1442 | 152 | 170 | + | 8 |
| TACAACAAACAACCTTACA | NGO1455 | 95 | 113 | - | 9 |
|  | NGO1456 | 230 | 248 | + | 9 |
| TTAGTTAAATATTATTTTT | NGO1485 | 142 | 160 | + | 8 |
| TTGAAAAGGAATGATTATA | NGO1487 | 124 | 142 | - | 8 |
| AATGATAAGGATTATTATT | NGO1496 | 42 | 60 | - | 7 |
| TAACATTATGATTAATATA | NGO1526 | 24 | 42 | - | 9 |
| TATAAGAAGTATCTTACTG | NGO1528 | 43 | 61 | - | 5 |
| TAAAACACAGATTATTTTC | NGO1539 | 32 | 50 | - | 8 |
| TGCTGAAACGGATTATTTA | NGO1551 | 181 | 199 | - | 8 |
| TGTTAAAAAAGATTGATGT | NGO1565 | 46 | 64 | - | 8 |
| TGTATGAATTATCCCTTTC | NGO1575 | 27 | 45 | - | 9 |
|  | NGO1576 | 177 | 195 | + | 9 |
| TCAAAATAAGGAACGTTTC | NGO1591 | 56 | 74 | - | 8 |
| TATTAAAAGAATTTATTTT | NGO1624 | 110 | 128 | - | 9 |
| TGCAATAATAAATTACTGC | NGO1624 | 135 | 153 | + | 8 |
| TTCAAAAGTCAATACTTGC | NGO1630 | 105 | 123 | + | 8 |
| TGAAAATAAAAAAATTGCC | NGO1631 | 37 | 55 | - | 8 |
| AAAACATAGAACGAGTTGC | NGO1649 | 139 | 157 | + | 8 |
| TGCATATTTGCATTAATCA | NGO1659 | 96 | 114 | + | 5 |
| TAATATAAGCGGCGGTATT | NGO1683 | 56 | 74 | + | 7 |
|  | NGO1684 | 185 | 203 | - | 7 |
| TAAAAATATGAAATTTAAA | NGO1695 | 54 | 72 | + | 8 |
| TAAAATCAAAATAATTTGA | NGO1699 | 59 | 77 | + | 8 |
|  | NGO1700 | 143 | 161 | - | 8 |
| CCATACAACTATATTTTTT | NGO1738 | 72 | 90 | + | 8 |
| TAACAGCACGCTCATTGTC | NGO1745 | 49 | 67 | - | 9 |
| TCAAATAAGAATCGTTATC | NGO1751 | 257 | 275 | + | 7 |
| AGAAAATATGAATTCGACC | NGO1753 | 194 | 212 | + | 8 |
| AATATCCAGAATGATTATT | NGO1754 | 97 | 115 | + | 9 |
| TTTAAAACGGAATTTTATA | NGO1783 | 1 | 19 | - | 7 |
| TACTACAGCATTCCTTATA | NGO1805 | 171 | 189 | - | 9 |
| TAAAAAAGGAAATAGATGA | NGO1810 | 62 | 80 | + | 8 |
| AAGAAAAAAGATGATTTTC | NGO1845 | 65 | 83 | - | 8 |
| TTTAAAAACGAAATGCCGT | NGO1850 | 62 | 80 | + | 8 |
| TGAAAAAAAGAATGTTAAA | NGO1857 | 214 | 232 | + | 8 |
| TATAAAGAAAAACATTTTA | NGO1890 | 220 | 238 | + | 8 |
| GTATATCAGTATTATCTTC | NGO1895 | 35 | 53 | + | 9 |
|  | NGO1896 | 66 | 84 | - | 9 |
| TTCAAAAGGGTATTATATG | NGO1911 | 185 | 203 | + | 8 |
| AGAAAATAAGATTTTTGAG | NGO1913 | 142 | 160 | - | 8 |
| TATAAAGAAGAAGGTTTGG | NGO1935 | 139 | 157 | + | 8 |
| TATAAAAAGAAAGTATTTT | NGO1948 | 87 | 105 | - | 7 |
| TATAAAAAGAAAGTATTTT | NGO1948 | 217 | 235 | - | 9 |
|  | NGO1949 | 96 | 114 | + | 9 |
| TAAAAGAAAACTCATTCTC | NGO1956 | 43 | 61 | + | 8 |
|  | NGO1957 | 250 | 268 | - | 8 |
| TATAAATAGAAAATATTGT | NGO1961 | 65 | 83 | - | 8 |
| TGTAAAATAGTATGGTTTT | NGO1961 | 132 | 150 | - | 7 |
|  | NGO1962 | 42 | 60 | + | 8 |
| TCAAAAAACTATATTTTTC | NGO1999 | 246 | 264 | - | 8 |
| AAGAAAACGGAATAGTTAT | NGO2026 | 51 | 69 | + | 7 |
| TAAAATAAGGTAAAGTTTT | NGO2029 | 18 | 36 | - | 9 |
| AGAAAACAGGAGCATTTTT | NGO2041 | 381 | 399 | + | 9 |
| TACAAATGCAAGTTATTTT | NGO2048 | 51 | 69 | + | 8 |
| TATATTATTAATCATCCTT | NGO2048 | 204 | 222 | - | 7 |
| AAAAATAATTATTATTATT | NGO2093 | 314 | 332 | + | 4 |
| TAAAAAATTTTTCGTTTTC | NGO2096 | 257 | 275 | + | 8 |
| CATAATAAGAACCGCTTTT | NGO2109 | 5 | 23 | - | 7 |
| AATAATAGTTTTGATTATC | NGO2111 | 182 | 200 | + | 9 |
| TTCATAAAGGGAATGTTTC | NGO2143 | 187 | 205 | - | 8 |
| TAAAATAAGGAGTATTTGA | NGO2170 | 116 | 134 | + | 7 |

*The two genes are divergently transcripted in the genome so that they share a same promoter region.
